# Photobiomodulation therapy for congenital color vision deficiency: results of a preliminary randomized clinical trial

**DOI:** 10.3389/fmed.2024.1497501

**Published:** 2024-12-19

**Authors:** Yidi Liu, Qinghua Yang, Jun Yu, Liang Jia

**Affiliations:** ^1^Department of Laser Medicine, the First Medical Center, Chinese PLA General Hospital, Beijing, China; ^2^Senior Department of Ophthalmology, the Third Medical Center, Chinese PLA General Hospital, Beijing, China; ^3^Department of Ophthalmology, Yiwu Aier Eye Hospital, Yiwu, China

**Keywords:** color vision deficiency, deutan, protan, photobiomodulation, phototherapy

## Abstract

**Purpose:**

This study presents a novel randomized controlled trial investigating photobiomodulation (PBM) therapy as an intervention method for color vision deficiency (CVD).

**Methods:**

A total of 74 participants with CVD were assigned to either the PBM group or the control group. In the PBM group, participants wore virtual reality (VR) goggles twice daily, with a 12-h interval, over a four-week period. The VR video consisted of alternating red and green images, each presented for 5 s, totaling 6 min and 20 s. No treatment was administered to the control group. Color vision improvement was assessed using Yu’s, Ishihara’s pseudoachromatic plates, Color Blindness Check (CBC), and FM-100 Hue total error score (TES).

**Results:**

After 4 weeks, in terms of Yu’s and Ishihara’s Plates, the patients in PBM group could identify increasing pieces (before: 1.6 ± 1.6, 2.3 ± 2.2; 4 weeks: 6.5 ± 4.4, 5.4 ± 2.9), while in control group, the number was before: 2.6 ± 3.4, 2.6 ± 2.5; 4 weeks: 3.3 ± 3.6, 2.9 ± 2.2. As for CBC scores, the patients in PBM also showed improved high scores (before: 2353.3 ± 700.0; 4 weeks: 2693.6 ± 642.5). Moreover, PBM treatment resulted in a significant reduction of FM-100 scores (before: 298.0 ± 211.3; 4 weeks: 202.1 ± 114.4).

**Conclusion:**

These findings suggest that PBM therapy holds promise as a potential treatment option for individuals with CVD.

**Clinical trial registration:**

The study received approval from the Ethics Committee of PLA General Hospital, China (KY2021-017). Additionally, it was registered as a Chinese domestic clinical trial (ChiCTR2200056761) at “http://Chictr.org.cn/index.aspx”.

## 1 Introduction

Color vision deficiency (CVD) is a congenital or acquired functional disorder that results in confusion and difficulty in recognizing colors (1). It is inherited in an X-linked recessive manner, leading to a higher prevalence of CVD in males compared to females (2). The reported prevalence of CVD varies across different geographical locations, ranging from 2 to 8% in boys (3–5) and 0.4–1.7% in girls (6). In China, the incidence rate was approximately 4.7% in boys and 0.7% in girls (7), indicating that more than 35 million people are affected by this disorder.

Individuals often remain unaware of their CVD until they undergo entrance examinations. According to the “Guiding Opinions on Physical Examination for Enrollment in Ordinary Colleges and Universities,” more than 50 professions are restricted to individuals with CVD. Consequently, people with CVD are excluded from certain professions such as the military, aviation, and specific medical fields (8, 9), due to their distinct disadvantage in performing visual tasks (10). As a result, CVD significantly impacts people’s daily lives, hindering them from achieving their goals and ambitions (11, 12).

In recent years, there has been increasing interest in the treatment of CVD, and extensive research has yielded significant progress. Gene therapy has shown promising results in non-human primates but remains untested in humans (13, 14). Additionally, individuals with CVD often rely on wearable devices to cope with everyday challenges. Tinted glasses or lenses are a common choice, with companies like Chromagen developing red contact lenses; however, their effectiveness varies among subjects (15, 16). Furthermore, smart goggles incorporating image processing algorithms, such as those developed by Google, have been used in CVD research, but their bulky size renders them impractical for everyday use. Therefore, it is crucial to discover a convenient, effective, and safe method to assist individuals with CVD in addressing their vision problems.

**Figure 1:**
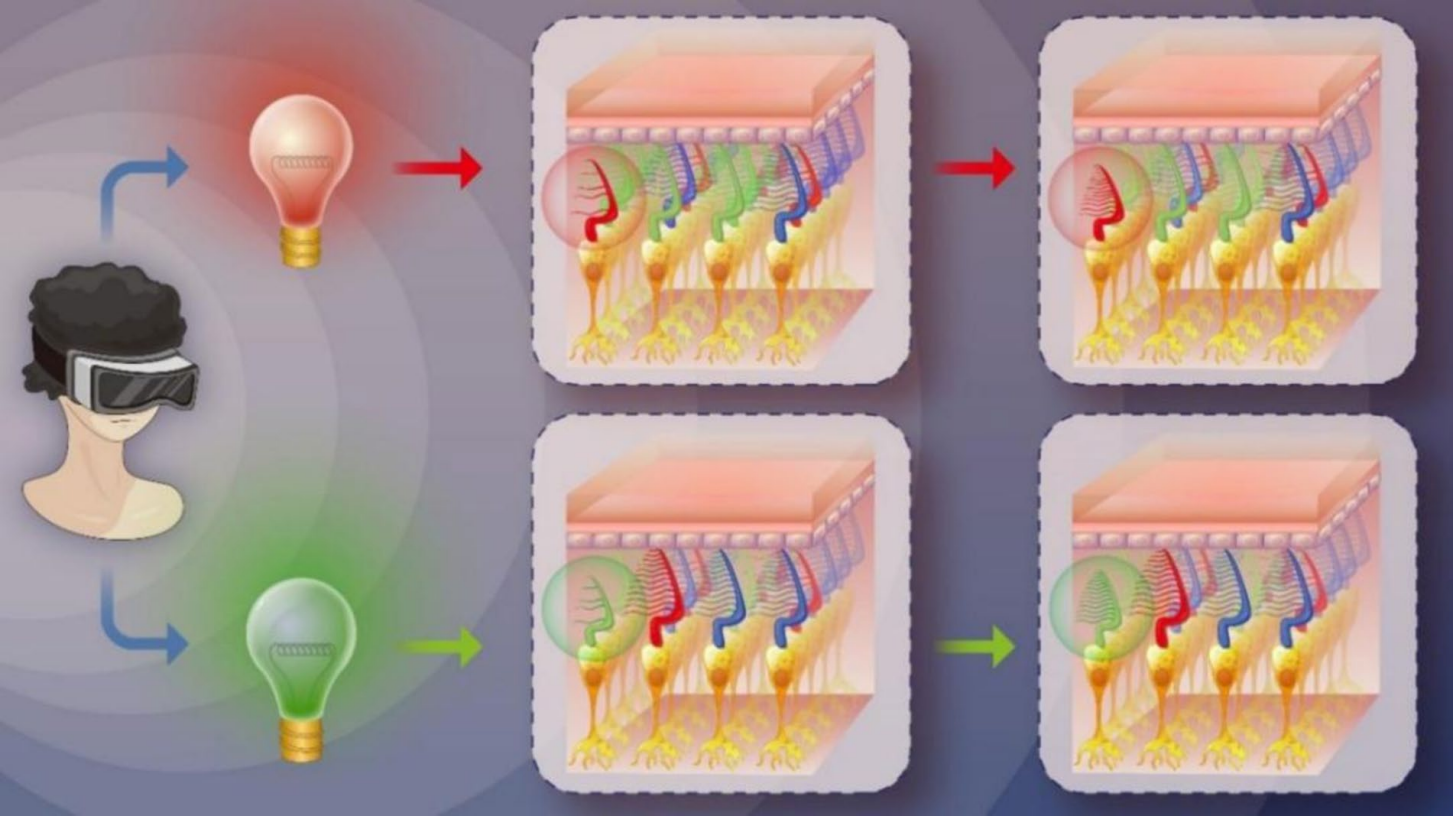
The assumption of PBM treating CVD.

Low-Level Light Therapy (LLLT) (17) a form of photobiomodulation (PBM) therapy, involves the therapeutic application of low-power visible and infrared light sources, such as lasers, LEDs, and broadband light. PBM is a non-thermal process that utilizes endogenous chromophores capable of absorbing light and inducing photochemical events. In recent years, PBM has been investigated for its potential to improve visual function, particularly in age-related macular degeneration (AMD). For instance, Grewal et al. utilized a handheld 670 nm light source with a power of 40 mW/cm^2^ for 2 min daily to treat AMD; although the results indicated insensitivity to the light source in AMD, the control group with aged eyes exhibited improved scotopic thresholds (18). Similarly, Shinhmar et al. demonstrated that illuminating the dominant eye with 670 nm LEDs at 40 mW/cm^2^ for 3 min every morning over 2 weeks enhanced photoreceptor performance (19). In 2021, Shinhmar et al. further conducted a study revealing that single 3-min exposures of 670 nm light (8 mW/cm^2^) in the morning significantly improved cone-mediated color contrast thresholds in aging populations, effectively restoring levels comparable to those observed in younger subjects for up to 1 week (20). These findings suggest that PBM can enhance rod and cone function. Motivated by these studies, we hypothesized that PBM could improve cone function in individuals with protan-deutan color blindness, which accounts for 95% of all CVD cases. Thus, we designed a novel alternating red-green light PBM schedule to treat individuals with this form of CVD (see Figure 1). To the best of our knowledge, this study is the first to utilize PBM for improving color discrimination.

## 2 Methods

### 2.1 Study design

From January to June 2022, participants were randomly assigned to either the PBM group or the control group. All subjects had confirmed cases of CVD but were unaware of the specific research objectives. The control group was recruited for the study without receiving any interventions.

**Sample Size**: No relevant studies have reported on the use of PBM for treating CVD. Considering the results of the preliminary experiment and the necessity to recruit as many subjects as possible, the improvement rate was set at 40% for the PBM group and 5% for the control group. Employing 1:1 matching and accounting for a 30% dropout rate, we calculated that each group would require 72 subjects to achieve a power of 0.90 and a significance level of 0.05 when evaluating the difference between the PBM and control groups using Pass 15 statistical software (NCSS, Kaysville, UT).

The study adhered to the principles of the Declaration of Helsinki and received approval from the Ethics Committee of PLA General Hospital, China (KY2021-017). Additionally, it was registered as a Chinese domestic clinical trial (ChiCTR2200056761) at Chictr.org.cn/index.aspx, and written informed consent was obtained from all participants.

### 2.2 Participants and color vision assessment

Subjects were recruited for this study via an advertisement seeking individuals with CVD to participate in an experiment. Accurate diagnosis of CVD involved a combination of medical history, comprehensive clinical examination, and multiple color vision tests. Specifically, clinical manifestations typical of this condition were taken into consideration during the diagnosis.

Pseudo-achromatic tests known as the Yu’s and Ishihara’s plates, developed in China and Japan, are utilized to assess an individual’s CVD status (21, 22). Nonetheless, individuals with CVD frequently struggle to differentiate between the colors on the Yu’s and Ishihara’s plates, thus experiencing difficulty in identifying the numbers.

The Color Blindness Check (CBC) is a test assessing an individual’s color vision using a mobile application downloadable from the Google Play Store.^1^

The FM-100 Hue Total Error Score (TEM) serves as an indicator of color vision, with higher scores indicating more severe color blindness.

Inclusion criteria required: (1) Visual acuity corrected to normal 20/25 or better; (2) Recognition score of less than 20 on the Yu’s or Ishihara’s plates (specifically 2nd to 16th, 37th to 40th, and 42nd for Yu’s plates, and 2nd to 17th, 22nd to 25th for Ishihara’s plates); (3) FM-100 TES score exceeding 50 (23).

Exclusion criteria: (1) Ability to correctly identify at least 20 Yu’s or Ishihara’s Plates; (2) FM-100 TES score of 50 or less (23); (3) CBC Severity equals 0.

**Figure 2:**
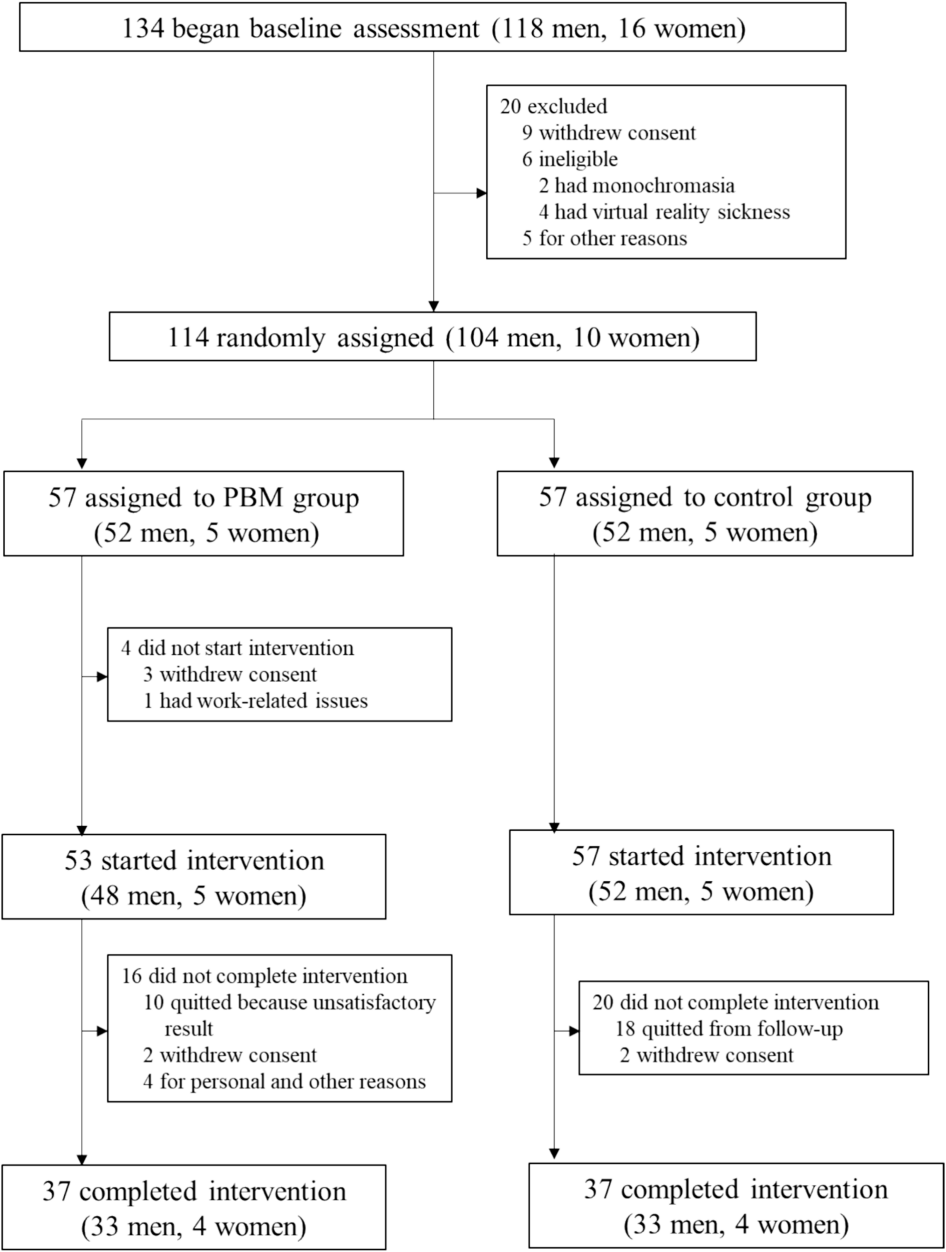
Trial profile.

The study profile for the enrolled subjects is depicted in Figure 2.

### 2.3 Procedures

Subjects in the control group received no treatment, while those in the PBM group underwent color vision training by watching a specially designed video (refer to Supplementary material S1) using virtual reality (VR) goggles twice a day, with a 12-h interval, over a duration of c During the VR goggle usage, all participants remained awake and in a resting state, with their eyes open and able to blink normally. Color vision assessments (using Yu’s plates, Ishihara’s plates, CBC, FM-100 TES) were conducted at the end of the first, second, and fourth week of PBM treatment. We specifically designed this video for subjects with protan and deutan defects.

**Figure 3:**
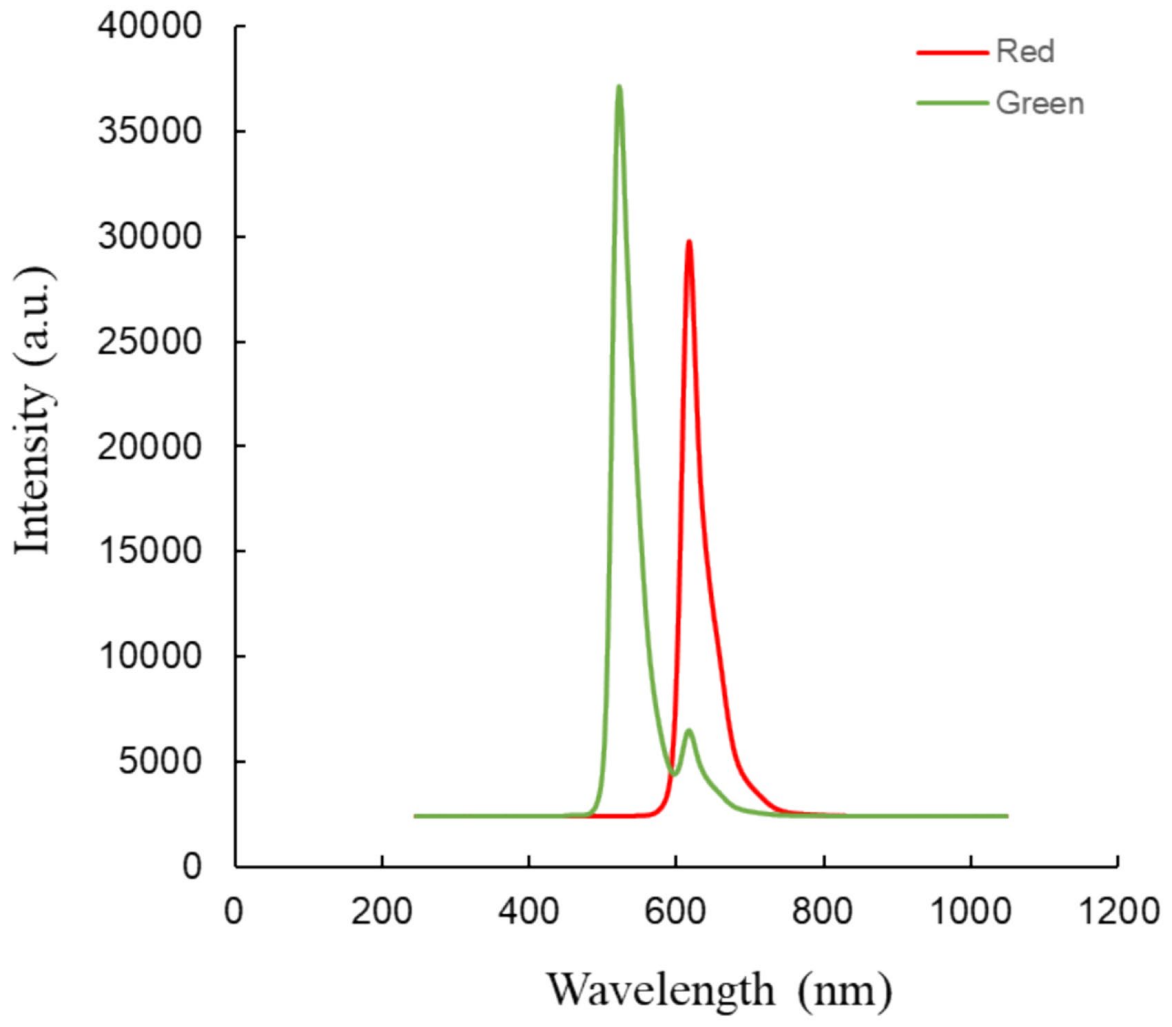
The wavelength of therapeutic light.

The video comprises two parts: red light irradiation and green light irradiation (refer to Figure 3). Each part lasted for 5 s and the entire video had a duration of 6 min and 20 s. The red light had a central wavelength of 621 nm with full-field illumination, and an energy density of approximately 8.69*10^−3^ W/m^2^ at the cornea. The green light had a central wavelength of 524 nm with full-field illumination, and an energy density of approximately 2.21*10^−2^ W/m^2^ at the cornea (24).

### 2.4 Data analysis

Baseline values were presented as Mean ± SD. Differences in baseline characteristics between groups were analyzed using Wilcoxon and Fisher exact tests. A repeated measures analysis of covariance (ANCOVA) was conducted using a mixed model, with the baseline change serving as the dependent variable and PBM treatment, time, and the interaction of treatment times as the independent variables. All statistical analyses were performed using SPSS software (version 26.0, SPSS, Inc.).

## 3 Results

### 3.1 Baseline of the recruited participants

Baseline assessment was initiated with 134 participants according to Figure 1, of whom 114 were subsequently randomized. Fifty percent (*n* = 57) were assigned to the PBM treatment group, while the remaining 50% (*n* = 57) were allocated to the follow-up-only control group. Among them, 64.9% (*n* = 37) in each group completed the study (Figure 1). Table 1 presents the baseline characteristics of the 74 enrolled participants. In both the control and PBM groups, 89.2% (*n* = 33) were male. The mean age was 27.2 ± 10.9 years in the control group and 27.8 ± 11.3 years in the PBM group. Regarding color vision defects, the control group had 35.1% (*n* = 13) with protan defects and 64.9% (*n* = 21) with deutan defects, while the PBM group had 27.0% (*n* = 10) with protan defects and 73.0% (*n* = 23) with deutan defects.

**Table 1:** Baseline of the recruited subjects.

	Control group	PBM group
Male (n, %)	33, 89.2%	33, 89.2%
Age	27.2 ± 10.9	27.8 ± 11.3
Protan defects (n, %)	13, 35.1%	10, 27.0%
Deutan defects (n, %)	21, 64.9%	23, 73.0%
Total	37	37

### 3.2 Effectiveness of PBM therapy in treating CVD

To evaluate the improvement of color vision in CVD subjects, we utilized five different methods: Yu’s plate recognition, Ishihara’s plates recognition, CBC scores, CBC severity scores, and FM-100 TES. Table 2 summarizes the effects of PBM treatment on CVD subjects at various time points (1 week, 2 weeks, and 4 weeks).

**Table 2:** PBM effect on CVD at different time points.

	Control group (*n* = 37)	PBM group (*n* = 37)	Between-group *p* value
Yu’s pseudoisochromatic plates			
Baseline	2.6 ± 3.4	1.6 ± 1.6	0.105
1 week	2.6 ± 3.4	3.7 ± 2.9	/
2 weeks	3.1 ± 3.9	4.9 ± 3.7	/
4 weeks	3.3 ± 3.6	6.5 ± 4.4	/
Change at one-week	0.6 ± 1.4	2.1 ± 2.0†	<0.0001
Change at two-week	0.5 ± 1.5	3.3 ± 2.8†	<0.0001
Change at four-week	0.7 ± 1.4	4.9 ± 3.6†	<0.0001
Ishihara’s pseudoisochromatic plates			
Baseline	2.6 ± 2.5	2.3 ± 2.2	0.625
1 week	2.8 ± 2.6	3.8 ± 2.1	/
2 weeks	3.0 ± 2.2	4.0 ± 2.4	/
4 weeks	2.9 ± 2.2	5.4 ± 2.9	/
Change at one-week	0.3 ± 1.5	1.5 ± 2.1§	<0.0001
Change at two-week	0.5 ± 1.6	1.7 ± 2.1§	<0.0001
Change at four-week	0.4 ± 1.2	3.1 ± 2.5†	<0.0001
CBC scores			
Baseline	2569.1 ± 762.8	2353.3.6 ± 3.4	0.215
1 week	2480.3 ± 793.2	2493.2 ± 672.5	/
2 weeks	2554.5 ± 774.7	2609.3 ± 641.5	/
4 weeks	2585.8 ± 764.9	2693.6 ± 642.5	/
Change at one-week	−88.8 ± 235.0	139.9 ± 256.5	<0.0001
Change at two-week	−14.6 ± 240.2	256.0 ± 262.3	<0.0001
Change at four-week	16.7 ± 203.4	340.3 ± 307.5*	<0.0001
CBC severity			
Baseline	57.0 ± 32.0	64.6 ± 25.7	0.271
1 week	60.1 ± 31.7	63.9 ± 26.3	/
2 weeks	58.2 ± 32.7	59.8 ± 25.1	/
4 weeks	57.6 ± 32.5	56.1 ± 26.0	/
Change at one-week	3.2 ± 8.8	−0.65 ± 9.7	<0.0001
Change at two-week	1.2 ± 8.0	−4.8 ± 10.7	<0.0001
Change at four-week	0.6 ± 7.3	−8.4 ± 11.9	<0.0001
FM-100 TES			
Baseline	234.6 ± 148.0	298.0 ± 211.3	0.145
1 week	248.9 ± 154.0	236.6 ± 146.2	/
2 weeks	244.9 ± 156.9	225.7 ± 155.1	/
4 weeks	242.4 ± 147.9	202.1 ± 114.4	/
Change at one-week	14.3 ± 54.4	−61.4 ± 127.3	<0.0001
Change at two-week	10.2 ± 52.5	−72.3 ± 150.7	<0.0001
Change at four-week	7.8 ± 34.7	−95.9 ± 153.4*	<0.0001

All *p* values reflect Bofferroni correction. *p < 0.05, §*p* < 0.01, ¶*p* < 0.001, †*p* < 0.0001 vs. baseline.

#### 3.2.1 Yu’s Pseudoisochromatic plates

**Figure 4:**
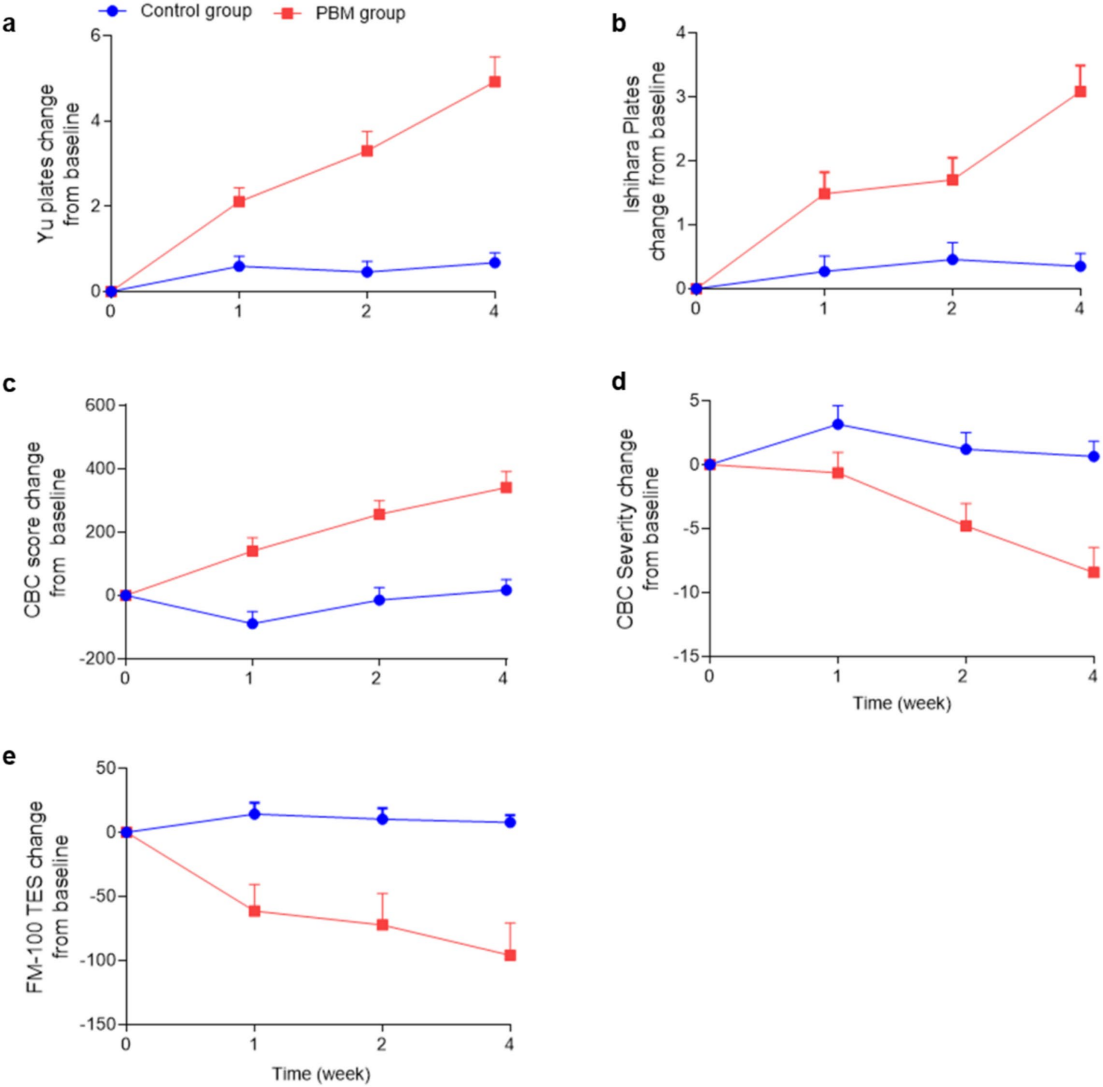
Color vision changes in the PBM and control groups. (“CBC” is an abbreviation for “Color Blindness Check”, “FM-100 TES” is an abbreviation for “FM-100 Hue Total Error Score”).

The effectiveness of PBM in enhancing subjects’ ability to recognize Yu’s plates is demonstrated in Figure 4A. The dependent variable, representing the effect of PBM, was calculated by subtracting the pre-treatment value from the post-treatment value. As depicted in Figure 4A and Table 2, PBM exhibited significant improvement in the recognition rate as early as 1 week after treatment, with a progressively increasing effect. After 4 weeks, the PBM group demonstrated the highest number of correct identifications (before: 1.6 ± 1.6; 1 week: 3.7 ± 2.9; 2 weeks: 4.9 ± 3.7; 4 weeks: 6.5 ± 4.4). Additionally, repeated ANOVA analysis revealed a positive correlation between treatment duration and the effectiveness of PBM (*p* < 0.05).

In the control group, it is evident that color vision did not improve over time without any treatment (before: 2.6 ± 3.4; 1 week: 2.6 ± 3.4; 2 weeks: 3.1 ± 3.9; 4 weeks: 3.3 ± 3.6). Furthermore, a statistically significant difference was observed between the two groups at each time point (*p* < 0.0001).

#### 3.2.2 Ishihara’s pseudoisochromatic plates

The effects of PBM on Ishihara’s pseudoisochromatic plates were clearly observed, as depicted in Figure 4B and Table 2. Following 1 week of PBM treatment, subjects demonstrated an improvement in identifying color fragments, with a mean of 3.8 ± 2.1 pieces compared to 2.3 ± 2.2 pieces before treatment. Notably, the effect of PBM increased over time (2 weeks: 4.0 ± 2.4; 4 weeks: 5.4 ± 2.9). Moreover, the PBM group achieved the highest recognition rate after 4 weeks, and repeated ANOVA analysis indicated a significant positive relationship between treatment duration and effectiveness (*p* < 0.05).

In contrast, the control group did not demonstrate any significant improvement in recognizing Yu’s plates (before treatment: 2.6 ± 2.5; 1 week: 2.8 ± 2.6; 2 weeks: 3.0 ± 2.2; 4 weeks: 2.9 ± 2.2). Moreover, a statistically significant difference was observed between the experimental and control groups at each time point except the baseline (*p* < 0.0001).

#### 3.2.3 CBC scores and CBC severity

The CBC scores serve as a reliable measure of the subjects’ color vision ability, where higher scores indicate superior performance. Following PBM treatment, the CBC scores exhibited a progressive increase from 2353.3 ± 700.0 to 2693.6 ± 642.5, as illustrated in Figure 4C and Table 2.

The CBC values of the control group exhibited no significant alterations throughout the study period (Before: 2569.1 ± 762.8; 1 week: 2480.3 ± 793.2; 2 weeks: 2554.5 ± 774.7; 4 weeks: 2585.8 ± 764.9, as presented in Table 2).

The severity of color blindness confusion (CBC) is determined based on the CBC scores, with higher severity indicating a reduced ability to perceive colors. Figure 4D and Table 2 illustrate a decreasing trend in CBC severity within the PBM group (Before: 64.6 ± 25.7; 1 week: 63.9 ± 26.3; 2 weeks: 59.8 ± 25.1; 4 weeks: 56.1 ± 26.0), while the control group exhibited no change (Before: 57.0 ± 32.0; 1 week: 60.1 ± 31.7; 2 weeks: 58.2 ± 32.7; 4 weeks: 57.6 ± 32.5).

The findings from repeated ANOVA analysis (*p* < 0.05) demonstrated the time-dependent impact on both CBC values and severity. Furthermore, PBM treatment exhibited a remarkable superiority over the control group in enhancing color vision at all time points (*p* < 0.0001).

#### 3.2.4 FM-100 TES

The FM-100 is an internationally recognized color vision test, with higher scores indicative of poorer color discrimination. Our study revealed that PBM treatment resulted in a significant reduction of FM-100 scores (Before: 298.0 ± 211.3; 1 week: 236.6 ± 146.2; 2 weeks: 225.7 ± 155.1; 4 weeks: 202.1 ± 114.4), whereas the control group remained unchanged throughout the study period (Before: 234.6 ± 148.0; 1 week: 248.9 ± 154.0; 2 weeks: 244.9 ± 156.9; 4 weeks: 242.4 ± 147.9), as depicted in Figure 4E and Table 2.

Repeated ANOVA analysis indicated that the duration of PBM treatment had a positive correlation with its effectiveness (p < 0.05). Furthermore, our findings demonstrated a significant difference in outcomes between the PBM and control groups at every time point (p < 0.0001).

#### 3.2.5 The effect of PBM on protan and deutan subjects

In our investigation, we sought to determine whether PBM had a differential effect on protan and deutan subjects. Table 3 presents a summary of the changes in five color vision assessments before and after PBM treatment. However, there was no statistically significant difference between protan and deutan subjects, indicating that PBM had the same effect on different types of CVD.

**Table 3:** Effects of PBM on individuals with protan and deutan color vision deficiencies.

	Protan group (*n* = 10)	Deutan group (*n* = 27)	Between-group *p* value
Yu’s pseudoisochromatic plates			
Baseline	1.2 ± 1.2	1.7 ± 1.7	0.371
Change at one-week	1.8 ± 2.2	2.2 ± 1.9	0.575
Change at two-week	3.3 ± 3.1	3.3 ± 2.6	0.997
Change at four-week	5.3 ± 4.1	4.8 ± 3.3	0.702
Ishihara’s pseudoisochromatic plates			
Baseline	1.3 ± 1.2	2.7 ± 2.3	0.095
Change at one-week	1.5 ± 1.8	1.5 ± 2.1	0.981
Change at two-week	1.6 ± 2.1	1.7 ± 2.1	0.861
Change at four-week	3.2 ± 3.3	3.0 ± 2.1	0.864
CBC scores			
Baseline	2116.0 ± 771.8	2441.1 ± 649.9	0.221
Change at one-week	134.1 ± 242.2	142.1 ± 261.5	0.935
Change at two-week	212.8 ± 275.4	272.0 ± 255.4	0.555
Change at four-week	289.3 ± 230.5	359.2 ± 329.5	0.552
CBC severity			
Baseline	72.8 ± 27.3	61.5 ± 24.5	0.248
Change at one-week	−3.6 ± 6.1	−0.4 ± 10.6	0.275
Change at two-week	−7.5 ± 8.5	−3.8 ± 11.2	0.361
Change at four-week	−7.5 ± 9.8	−8.8 ± 12.6	0.780
FM-100 TES			
Baseline	354.4 ± 249.6	277.1 ± 191.1	0.337
Change at one-week	−90.9 ± 74.8	−50.4 ± 140.4	0.405
Change at two-week	−78 ± 151.3	−70.2 ± 150.5	0.893
Change at four-week	−143.6 ± 169.8	−78.3 ± 142.9	0.262

All *p* values reflect Bofferroni correction. *p < 0.05, §*p* < 0.01, ¶*p* < 0.001, †*p* < 0.0001 vs. baseline.

Our study aimed to investigate whether PBM had a differential effect on protan and deutan subjects. Table 3 summarizes the changes in five color vision assessments before and after PBM treatment. Our findings indicate that there was no statistically significant difference between the two groups, suggesting that PBM had a similar effect on both types of CVD.

## 4 Discussion

Normal color vision is mediated by cones containing blue-, green-, and red-sensitive opsin pigments, often referred to as short (S, 426 nm, blue), medium (M, 530 nm, green), and long (L, 557 nm, red) wavelength-sensitive pigments (25).

Deuteranomalous and protanomalous deficiencies are characterized by a greater degree of overlap in spectral sensitivity than normal, with peak sensitivity shifts occurring in green-sensitive and red-sensitive pigments, respectively (25). In deuteranomaly, the normal M cone is replaced by an abnormal cone, denoted as L’, with spectral sensitivity close to that of the normal L cone. Similarly, in protanomaly, the L cone is replaced by M’, with spectral sensitivity close to that of the normal M cone. Deuteranomalous and protanomalous deficiencies are characterized by a significant spectral overlap between the sensitivities of the middle and long wavelength cone types, leading to a reduction in chromatic contrast within the corresponding region of the visible spectrum. Consequently, color discrimination is impaired (26, 27).

Our study shows the efficacy of photobiological modulation (PBM) in treating color vision deficiencies (CVD). Following 1 week of PBM therapy, deuteranomaly and protanomaly patients demonstrated significant improvement in color vision, with increased response rates to pseudoisochromatic plates and CBC, and decreased FM100 TES and severity scores, compared to the control group. Furthermore, PBM treatment demonstrated sustained efficacy with longer treatment durations of up to 4 weeks.

Photobiological modulation (PBM) is a novel approach that utilizes photon energy to regulate cellular and tissue function and correct chromatic aberrations. This preliminary study investigated the efficacy of PBM in improving color vision deficiencies in subjects with weak red or green color perception. Red light (621 nm) with an energy density of 8.69*10^−3^ W/m^2^ and green light (524 nm) with an energy density of 2.21*10^−2^ W/m^2^ were applied twice daily for 6 min and 20 s. The study demonstrated significant improvement in color vision ability, as evidenced by an increased number of plates recognized on the Yu’s and Ishihara’s tests after 2 and 4 weeks of treatment. Upon literature review and inference of the possible mechanism, there is evidence indicating a significant increase in the gain of the S-(L + M) mechanism between the retina and cortex (28, 29). This suggests that the function or number of S, L, and M cones undergoes dynamic changes. These adjustments likely take place during natural adaptation processes that calibrate sensitivity and neural gain based on environmental stimuli (30–32), optimizing the utilization of neurons’ limited response range (33). Following exposure to red (621 nm) and green (524 nm) light, the L’ cones converted into normal M cones, and the M’ cones transformed into normal L cones, suggesting adaptive adjustments.

There are still some limitations existed in our study. Our study is relatively preliminary and the core purpose is to find whether PBM is effective in treating CVD. In control group, the patients included receive no treatment, however, the more proper way is letting patients watch white light video which lasts the same time as treatment group. Moving forward, the tests of the optimum wavelength, light intensity, duration of PBM should be further designed to be sure. The underlying mechanism of PBM is also crucial, which need to be delved deeper.

PBM represents a safe, convenient, and cost-effective therapeutic approach for managing CVD. Our study has effectively showcased the untapped potential of PBM, instilling confidence that it holds tremendous benefits for a wider population afflicted by CVD.

## 5 Conclusion

PBM applies light of low density on the lesion tissue or monolayer cells to cause non-destructive and non-thermal biological reactions for therapeutic purposes. In this study, red light (621 nm) with an energy density of 8.69*10^−3^ W/m^2^ and green light (524 nm) with an energy density of 2.21*10^−2^ W/m^2^ were applied twice daily for 6 min and 20 s. Following 1 week of PBM therapy, deuteranomaly and protanomaly patients demonstrated significant improvement in color vision. PBM treatment presented sustained efficacy of up to 4 weeks. However, our study represents an initial investigation into the comparative effectiveness of PBM. Moving forward, we aim to ascertain the optimal wavelength and duration of PBM, while also expanding our participant pool. Furthermore, it is crucial to figure out the underlying mechanism of PBM.

## Data Availability

The original contributions presented in the study are included in the article/Supplementary material, further inquiries can be directed to the corresponding authors.

## Footnotes

^1^ https://play.google.com/store/apps/details?id=ch.colblindor.colorblindcheck

## Supplementary material

SUPPLEMENTARY MATERIAL S1 The treatment video.
